# Brain function effects of exercise interventions for cognitive decline: a systematic review and meta-analysis

**DOI:** 10.3389/fnins.2023.1127065

**Published:** 2023-05-16

**Authors:** Diana Karamacoska, Ali Butt, Isabella H. K. Leung, Ryan L. Childs, Najwa-Joelle Metri, Vithya Uruthiran, Tiffany Tan, Angelo Sabag, Genevieve Z. Steiner-Lim

**Affiliations:** ^1^NICM Health Research Institute, Western Sydney University, Penrith, NSW, Australia; ^2^Translational Health Research Institute (THRI), Western Sydney University, Penrith, NSW, Australia; ^3^School of Health Sciences, Western Sydney University, Campbelltown, NSW, Australia; ^4^School of Medicine, Western Sydney University, Penrith, NSW, Australia; ^5^Discipline of Exercise and Sport Science, Faculty of Medicine and Health, The University of Sydney, Sydney, NSW, Australia

**Keywords:** dementia, mild cognitive impairment, subjective cognitive decline, physical activity, exercise, cognition, Alzheimer's disease

## Abstract

**Introduction:**

Exercise is recognized as a modifiable lifestyle factor that can mitigate cognitive decline and dementia risk. While the benefits of exercise on cognitive aging have been reported on extensively, neuronal effects in adults experiencing cognitive decline have not been systematically synthesized. The aim of this systematic review was to assess the effects of exercise on cognition and brain function in people with cognitive decline associated with dementia risk.

**Method:**

A systematic search was conducted for randomized controlled trials of ≥ 4 weeks exercise (aerobic, resistance, or mind-body) that assessed cognition and brain function using neuroimaging and neurophysiological measures in people with subjective or objective cognitive decline. Study characteristics and brain function effects were narratively synthesized, while domain-specific cognitive performance was subjected to meta-analysis. Study quality was also assessed.

**Results:**

5,204 records were identified and 12 unique trials met the eligibility criteria, representing 646 adults classified with cognitive frailty, mild or vascular cognitive impairment. Most interventions involved 40-minute sessions conducted 3 times/week. Exercise improved global cognition (g = −0.417, 95% CI, −0.694 to −0.140, *p* = 0.003, *I*^2^ = 43.56%), executive function (g = −0.391, 95% CI, −0.651 to −0.131, *p* = 0.003, *I*^2^ = 13.28%), but not processing speed or general short-term memory (both *p* >0.05). Across fMRI and ERP studies, significant neuronal adaptations were found with exercise *cf*. control throughout the brain and were linked with improved global cognition, memory, and executive function. Cerebral blood flow was also found to improve with 24 weeks of exercise, but was not linked with cognitive changes.

**Discussion:**

The cognitive improvements associated with exercise are likely driven by increased metabolic activity, cerebrovascular mechanisms, and neuroplasticity throughout the brain. Our paper shows the promise in, and need for, high-quality trials integrating cognitive and brain function measures to elucidate the functional relationship between exercise and brain health in populations with a high risk of dementia.

**Systematic review registration:**

PROSPERO, identifier: CRD42022291843.

## 1. Introduction

Dementia is associated with a decline in cognition including memory, executive function, attention, language, thinking, and/or visuospatial skills that impacts functioning (Arvanitakis et al., 2019). As of 2020, ~55 million people have dementia worldwide, and this figure is expected to reach 152 million within the next 30 years (Martin et al., 2015). Alzheimer's disease (AD) is the most common type of dementia, contributing to an estimated 50–75% of all cases (Gallaway et al., 2017).

Although AD is frequently pathologically characterized by the accumulation of extracellular Aβ plaques (Hardy and Higgins, 1992), other proteinopathies are often present including neurofibrillary tangles of intracellular hyperphosphorylated tau protein, aggregates of TAR DNA-binding protein 43 (TDP-43), and α-synuclein pathology as seen in other causes of dementia (Josephs et al., 2014; DeTure and Dickson, 2019). Further, cerebrovascular dysfunction and injury commonly occurs in AD, leading to “mixed dementia”, a combination of AD and vascular pathologies (Craft, 2009; Govindpani et al., 2019). Together, this variable and multilayered pathology creates a neurotoxic environment associated with neuroinflammation, oxidative stress, excitotoxicity, and cell death (Coles et al., 2022). Given the increasingly blurred line between the traditional aetiological types of dementia, interventions with multitargeted effects—such as exercise—that can transcend these diagnostic entities and reduce dementia risk should be prioritized. Importantly, approximately 40% of dementia risk is thought to be modifiable, providing opportunity for risk reduction strategies that may reduce dementia incidence and/or delay deterioration (Paulson and Igo, 2011; Livingston et al., 2020).

The clinical syndrome for dementia occurs after a long-lasting asymptomatic preclinical phase followed by a symptomatic prodromal phase known as mild cognitive impairment (MCI) (Jack et al., 2010; Sperling et al., 2011). MCI is characterized by objective cognitive decline on the background of relatively intact activities of daily living (Petersen et al., 1999). Subjective cognitive decline (SCD), the self-reported worsening of cognition despite falling within the normal range of cognitive ability on neuropsychological tests (Molinuevo et al., 2017; van Harten et al., 2018; Jessen et al., 2020). Individuals with SCD and MCI have increased presence of AD biomarkers, cerebrovascular pathology, inflammatory burden (Blom et al., 2019; Shen et al., 2019), and an increased risk of developing dementia: ~2 fold for SCD and ~5 fold for MCI (Campbell et al., 2013; Perrotin et al., 2017; Pike et al., 2021). Vascular contributions to dementia risk, such as stroke and dysregulated cerebral blood flow, have also emerged as important markers for intervention efforts (Rundek et al., 2022). This makes the preclinical stages of dementia, including SCD, MCI, and vascular cognitive impairment (VCI), the ideal stages at which to intervene to try and prevent future cognitive decline, disability, and deterioration (Molinuevo et al., 2017; van Harten et al., 2018).

Physical activity including exercise is an important lifestyle factor that, when performed regularly, can decrease the risk of cognitive decline and dementia by 38% and 28%, respectively (Middleton et al., 2010; Sofi et al., 2011). Current guidelines, in Australia for example, suggest that older adults conduct physical activity of moderate intensity for 30 minutes on most, preferably all, days (Department of Health and Aged Care, 2021). Problematically, only 28% of Australian adults aged over 65 years currently meet these guidelines (Australian Institute of Health Welfare, 2022). It is well reported that exercise (aerobic, resistance or tai-chi) can improve cognition in older adults with and without cognitive decline (Snowden et al., 2011; Northey et al., 2018), particularly when combined with cognitive training (Cammisuli et al., 2017; Karssemeijer et al., 2017; Meng et al., 2022). Exercise is theorized to reduce cognitive deterioration risk through cerebrovascular improvements (Bliss et al., 2021), structural brain changes (Erickson et al., 2011; Bolandzadeh et al., 2015; ten Brink et al., 2015), and neural adaptations that stimulate neurogenesis and decrease cellular damage and pro-inflammatory responses (Vecchio et al., 2018). The functional brain changes associated with physical exercise in people experiencing cognitive decline that is associated with dementia risk are, however, less understood (Cui et al., 2018).

Functional brain measures can provide additional insights into the effects of exercise on the brain and cognitive function beyond that of structural brain measures (e.g., volumetric MRI; e.g., ten Brink et al., 2015), such as electrocortical (EEG, MEG, TMS), metabolic (PET, MRS), and cerebrovascular (fMRI, fNIRS, SPECT, ASL, TCD) activity (see Table 1; Steiner et al., 2017). Their application in exercise interventions in people with MCI has been growing, but with mixed effects being reported. For example, improvements in neuronal efficiency, as measured by fMRI, EEG, and PET, were identified in observational studies (Huang et al., 2016) while non-significant fMRI changes were found across RCTs (Ji et al., 2021). A systematic synthesis of RCTs is needed to understand the isolated effects of physical exercise on measures of cognition and brain function in people experiencing cognitive decline.

**Table 1:** Search strategy used to identify articles in EBSCOHost.

**Number**	**Search items**
#1	[Abstract] age-associated memory OR age-related memory OR age associated memory OR age related memory OR cognitive decline OR cognitive impairment OR MCI OR SCD OR neurocogniti^*^ OR preclinical dementia
#2	[Abstract] exercise OR physical activity OR walking OR running OR jogging OR cycling OR aerobic^*^ OR plyometric^*^ OR resistance training OR strength training OR weight training OR interval training OR yoga OR tai-chi OR tai chi
#3	[Abstract] cogniti^*^ OR memory OR attention OR executive function OR neuro^*^ OR electroencephalogram OR EEG OR event related potential OR ERP OR functional magnetic resonance imag^*^ OR fMRI OR transcranial magnetic stimulat^*^ OR TMS OR functional near-infrared spectroscopy OR fnirs OR positron emission tomograph^*^ OR PET OR CBF OR cerebral blood flow OR cerebral perfusion OR transcranial Doppler OR TCD OR arterial spin labeling OR ASL OR MEG OR magnetoencephalography OR Single photon emission computed tomography OR SPECT OR magnetic resonance spectroscopy OR MRS
#4	[Abstract] random^*^ control^*^ trial OR random^*^ control^*^ intervention OR random^*^ control^*^ study OR random^*^ clinical trial
#5	#1 AND #2 AND #3 AND #4

The aim of this systematic review was to address the research question: “*what are the study characteristics and effects of exercise interventions, on cognition and brain function in individuals with subjective or objective cognitive decline associated with dementia risk?”* The findings from this review can further knowledge on the mechanisms by which physical activity interventions may exert previously reported positive effects on cognition and brain function in people with high dementia risk.

## 2. Materials and methods

This systematic review was prospectively registered with PROSPERO (CRD42022291843). The conduct and reporting of this systematic review adhered to the Preferred Reporting Items for Systematic Reviews and Meta-Analyses (PRISMA) guidelines (Moher et al., 2009).

### 2.1. Eligibility criteria

To formulate and refine the eligibility criteria and search strategy, a scoping review was performed. Existing literature was reviewed to identify the population, intervention, comparisons, outcome, and study design (PICOS) principles used in this systematic review.

- **Population:** Adults classified as having either subjective or objective cognitive impairment associated with dementia risk (e.g., SCD, MCI, VCI).
- **Intervention:** Any structured form of exercise (aerobic training, resistance/strength training, mind-body exercises that involve movement) as the primary focus of the study.
- **Comparisons:** A control group consisting of a wait-list group, usual care, or inactive/static exercise (e.g., stretching).
- **Outcomes:** Any validated assessment of cognition (e.g., Mini Mental State Exam [MMSE], Montreal Cognitive Assessment [MoCA]) AND measure of brain function (functional magnetic resonance imaging [fMRI], electroencephalography [EEG], event-related potentials [ERPs], positron emission tomography [PET], functional near-infrared spectroscopy [fNIRS], transcranial magnetic stimulation [TMS]), cerebral blood flow [CBF], cerebral perfusion, transcranial Doppler [TCD], arterial spin labeling [ASL], magnetoencephalography [MEG], single photon emission computed tomography [SPECT], magnetic resonance spectroscopy [MRS]).
- **Study designs:** Randomized controlled trial design of ≥ 4 weeks in duration to explore the effects of chronic exercise (Farrell and Turgeon, 2023).

To be eligible for inclusion, articles must have been peer-reviewed and published in English, without any limitation on publication year. Cognition and brain function assessments must have been conducted pre/post intervention. Cognitive measures were deemed valid based on whether an appropriate citation for the assessment tool was provided within the reported study (as assessed in the methodological quality checklist described in Section 2.5). Measures of brain function specifically related to neuroimaging and physiological measurement modalities that quantify the functional activity of the brain including fMRI, EEG, MEG, PET, SPECT, fNIRS, TMS, MRS, TCD, and ASL. Studies only reporting structural brain measures (e.g., volumetric MRI, diffusion weighted imaging) were excluded. If a study involved multiple groups of the same exercise type (e.g., resistance training once/week in group one and resistance training twice/week in group two), the protocol that most closely adhered to current physical activity guidelines (Bull et al., 2020) was assessed further. Studies were excluded if: a full text could not be obtained or they were published in a format other than a journal article (e.g., review, book chapter, opinion article, thesis or editorial). Studies that did not measure the isolated effects of exercise were excluded (e.g., multimodal studies and exergaming), as were studies which included people with comorbid neurological (e.g., Parkinson's disease) or psychiatric conditions (e.g., depression).

### 2.2. Systematic search strategy

We conducted a search for articles published in EBSCOHost (indexing CINAHL Plus, SportDiscus, PsycINFO, APA PsycArticles, Psychology and Behavioral Sciences Collection, and Ageline), Web of Science (indexing Web of Science Core Collection, Current Contents Collection, Chinese Science Citation Database, KCI-Korean Journal Database, MEDLINE, SciELO Citation Index) from inception to 10 March 2023. Search terms included words related to our PICOS. Table 1 shows the advanced search strategy used in EBSCOHost, which was adapted for the other database.

### 2.3. Study selection and data extraction

All retrieved study titles and abstracts were imported into COVIDENCE. Following the removal of duplicates, titles and abstracts were independently screened by two researchers (RC and NJM) and full texts of potentially relevant studies were obtained. The full texts of relevant studies were reviewed independently by three researchers (AS, GZS, and DK); this included the review of reference lists for potentially relevant articles. Study characteristics were extracted from the included articles by two researchers (VU and AB). Any disagreement about the selection of studies, or extraction of data, was resolved by discussion with one researcher (DK). Authors were contacted for additional information where needed. The following data were extracted from the studies: first author, publication year, location, study population, study design, number of participants (intervention and controls), cognitive decline or impairment classification criteria (if any), age (mean and SD), percentage of females included, and intervention characteristics (type, frequency, intensity [if reported], session duration, intervention duration), pre/post-intervention cognitive outcome data (mean, SD), and summary of brain function findings (between- and within-group effects, and *p* values). Two researchers (DK and GZS) categorized domain-specific cognitive performance according to the Cattell-Horn-Carroll and Miyake framework (Webb et al., 2018), and consensus was reached with a third researcher (IHKL).

### 2.4. Data synthesis

Cognitive outcome data are presented as effect sizes with 95% confidence intervals (CI). Where data from ≥3 studies were available for a given cognitive domain, random-effects meta-analyses were conducted using Comprehensive Meta-Analysis Version 3 software (Biostat Inc., Englewood, NJ, USA). Significance was set at *p* < 0.05. Effect sizes were calculated from pre- to post-intervention scores between two groups for change in cognition outcomes such as global cognition, executive function, processing speed, and general short-term memory and expressed as Hedge's *g* with 95% CIs around the estimated effect size. Absolute between-study heterogeneity was calculated using tau (τ). If post intervention scores were not available, the absolute or relative mean change scores were used to calculate effect sizes. Where there were two comparisons from the same study for a particular outcome (e.g., an aerobic exercise group and dancing group), the sample size of the control group was halved.

Statistical heterogeneity between studies was quantified using Cochran's Q and *I*^2^ statistics, both of which provide estimates of the degree of heterogeneity resulting from between-study variance, rather than by chance. Cochran's Q with *p* < 0.05 was classified as significant heterogeneity, and *I*^2^ of more than 75% was considered to indicate high level heterogeneity, *I*^2^ of 25%−75% as indicative of substantial heterogeneity, and an *I*^2^ < 25% as low heterogeneity. Publication bias was tested using the Begg and Mazumdar test, with a *p* < 0.05 suggesting the presence of bias (Sterne et al., 2019). Where significant bias was detected, a Duval and Tweedie trim-and-fill analysis (Duval and Tweedie, 2000) was conducted to re-calculate the pooled effect size after removing any studies which may introduce publication bias (i.e., small studies with large effect sizes from the positive side of the funnel plot). Brain function effects were narratively analyzed due to heterogenous outcomes and insufficient data available for meta-analysis.

### 2.5. Methodological quality

Study quality was assessed independently by two researchers (TT and AB) using a modified Downs and Black checklist (Downs and Black, 1998). Reporting quality is assessed with 10 items spanning the aims, participant characteristics, and findings; external validity is checked with 3 items concerning representativeness; internal validity for bias and confounding/selection bias with 4 and 6 items respectively. The scale was modified to include criteria on exercise supervision, whereby if a study was supervised, a “yes” was given. If an item was unable to be determined a “no” was given. Scores were compared and disagreements resolved by a third reviewer (AS).

### 2.6. Risk of bias

Studies were assessed for bias independently by two reviewers (TT and RLC) using the Cochrane Risk of Bias 2 tool (Sterne et al., 2019). Included studies were evaluated as having ‘low,' ‘high,' or ‘unclear' risk of bias using the following domains: randomization, allocation concealment, blinding of intervention instructors, blinding of participants, blinding of outcome assessors, handling of incomplete data, selective reporting, and other risk of bias pertaining to exercise adherence. Discrepancies in ratings were discussed and resolved with one additional reviewer (AS). Studies were not excluded based on their bias assessment.

## 3. Results

### 3.1. Study selection

**Figure 1:**
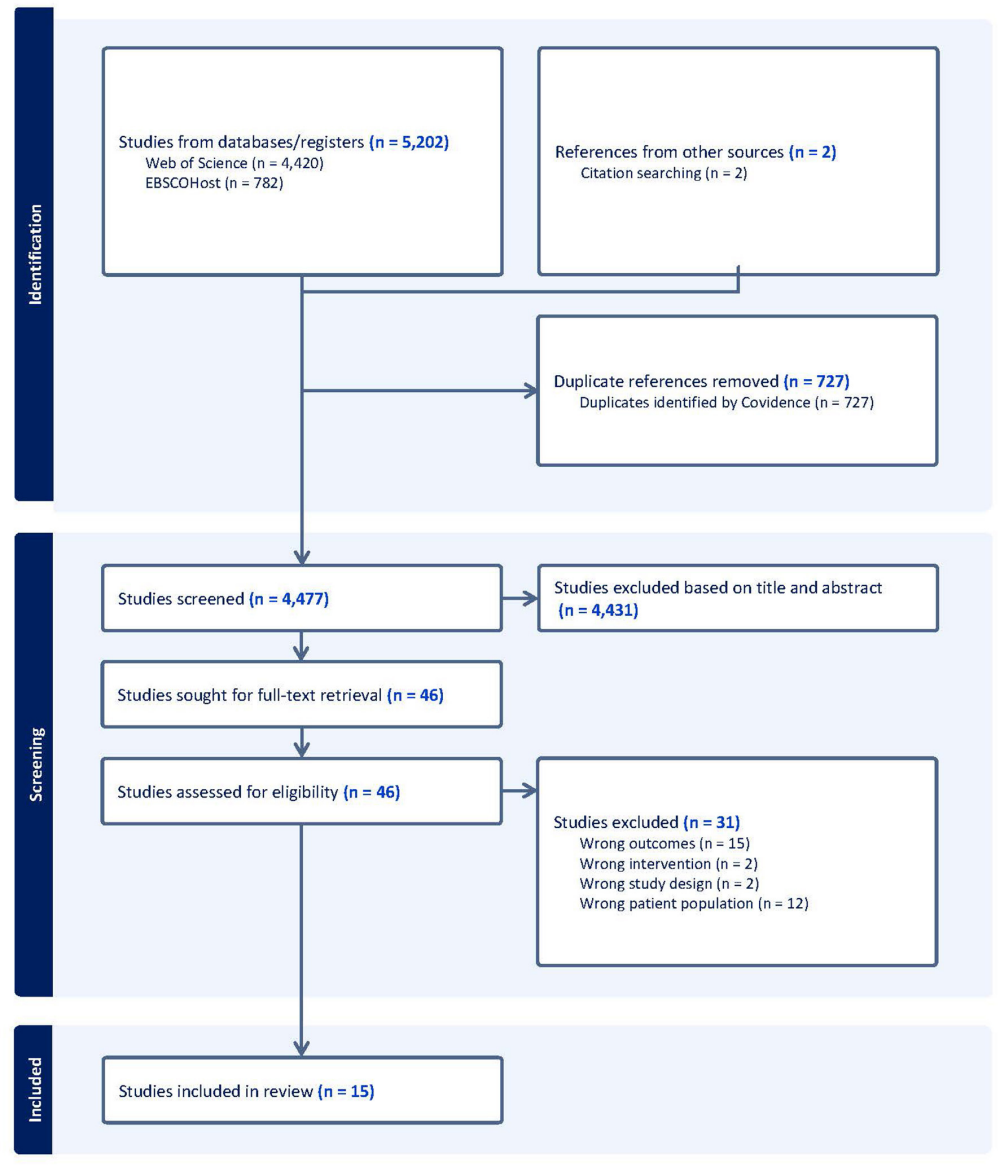
Flow chart of the study selection process.

Figure 1 represents the study selection process for this review. A total of 5,204 records were identified with 4,477 remaining after duplicates were removed. Fifteen articles met eligibility criteria for this systematic review, representing 12 unique interventions.

### 3.2. Study characteristics

The characteristics of the fifteen articles are shown in Table 2. There were three multi-paper interventions: two articles reported on different brain function outcomes from the one intervention (Zhu et al., 2018; Qi et al., 2019), two papers reported on the same intervention outcomes at different time-points (Suo et al., 2016 for intervention effects; Broadhouse et al., 2020 for follow-up), and two publications reported on different aspects of the same brain function measure from the one intervention (Tao et al., 2019; Lin et al., 2023). As we were interested in intervention effects here, the follow-up findings from Broadhouse et al. were excluded from further analysis, but are reported in Table 2 for interested readers. Further, as Lin et al. and Tao et al. referred to the same sample between their studies, only Tao et al.'s data was used in the meta-analysis. All articles were published between 2012 and 2023, with five studies located in China, two each in Canada and Australia, and one each in Israel, Pakistan, Taiwan, United States of America, and South Korea. Nine of the twelve unique interventions employed a two-group parallel arm design and the remaining four trials involved three groups where two exercise-related interventions were compared to a control.

**Table 2:** Study characteristics of the 15 identified exercise trials in people with cognitive impairment associated with increased dementia risk.

**References; location**	**Population; classification criteria**	**Group n**	**Age ±SD (years)**	**Female (%)**	**Study Arms (intervention description)**	**Outcome measures**
**Two-Arm RCTs**						
Amjad et al. (2019); Pakistan	MCI; Clinician's diagnosis with MMSE and MoCA <25	Intervention *n* =21	58.2 ± 2.3	47.6	Arm 1 (aerobic exercise): supervised at 60–80% max HR using a treadmill and stationary bicycle, gradually increasing from 20 to 40 mins, with 5–10 min warm-up and cool-down periods, 3 times/week for 6 weeks	Cognition: MoCA, Trail making test. Brain function: resting state EEG
		Comparison *n* =19	59.6 ± 2.7	47.4	Arm 2 (control): instructed to perform gentle movements and body stretching at home, with follow-up for adherence, for 3 times/week for 6 weeks	
Hsu et al. (2018); Canada	VCI; Erkinjuntti Criteria with MMSE >20 & MoCA <26	Intervention *n =* 19	72.6 ± 8.4	60.0	Arm 1 (aerobic exercise): 40 min supervised walking at 60–70% HR reserve, with 10 min warm-up and cool-down periods, 3 times/week for 24 weeks plus monthly VCI and diet educational material	Cognition: MoCA, flanker task. Brain function: task-based fMRI
		Comparison *n =* 19	72.5 ± 8.9	63.6	Arm 2 (control): received usual care plus monthly contact, and VCI and diet educational material, for 24 weeks	
Qi et al. (2019); China^*^	MCI; NIA-AA Criteria with MMSE >25 & MoCA ≤ 26	Intervention *n =* 16	70.6 ± 6.2	75.0	Arm 1 (aerobic exercise): 25 min supervised dance at 60–80% of max HR, with 5 min warm-up and cool-down periods, 3 times/week, plus usual care, for 12 weeks	Cognition: MoCA, trail making test, digit span. Brain function: resting state fMRI
		Comparison *n*= 16	69.1 ± 8.1	68.8	Arm 2 (control): received usual care for 12 weeks	
Tomoto et al. (2021); USA	Amnestic MCI; ADNI modified Petersen Criteria with MMSE 24-30	Intervention *n =* 22	64.8 ± 6.4	54.5	Arm 1 (aerobic exercise): 25–35 min supervised treadmill walking at 75–90% of max HR, with 5 min warm-up and cool-down periods, 3 times/week for 52 weeks	Cognition: MMSE, D-KEFS Trail Making Test. Brain function: Cerebral blood flow
		Comparison *n =* 30	66.1 ± 6.8	53.3	Arm 2 (control): 25–35 min supervised stretching at <50% of max HR, 3 times/week for 52 weeks	
Yogev-Seligmann et al. (2021); Israel	Amnestic MCI; NIA-AA Criteria	Intervention *n =* 13	70.8 ± 5.5	38.4	Arm 1 (aerobic exercise): 40 min supervised stationary bicycling at 70-80% of HR reserve, with 5 min warm-up and cool-down periods, 3 times/week for 16 weeks	Cognition: MoCA, Digit span. Brain function: task-based fMRI
		Comparison *n =* 14	71.9 ± 6.4	50.0	Arm 2 (control): 40 min supervised balance and tone exercises at <30% HR reserve, 3 times/week for 16 weeks	
Zhu et al. (2018); China^*^	MCI; NIA-AA Criteria with MMSE >25 & MoCA ≤ 26	Intervention *n =* 29	70.3 ± 6.7	51.7	Arm 1 (aerobic exercise): 25 min supervised dance at 60–80% of max HR, with 5 min warm-up and cool-down periods, 3 times/week, plus usual care, for 12 weeks	Cognition: MoCA, trail making test, digit span. Brain function: event-related potential
		Comparison *n =* 31	69.0 ± 7.3	67.7	Arm 2 (control): received usual care for 12 weeks	
Hong et al. (2018); South Korea	MCI; Petersen Criteria with K-MoCA <24	Intervention *n =* 10	78.0 ± 5.0	70.0	Arm 1 (resistance training): 40 min supervised exercises with an elastic band at 15 max repetition for 40 mins, with progressive increases in intensity from 65% intensity 1RM, and 10 min warm-up and cool-down periods, 2 times/week for 12 weeks	Cognition: K-MoCA, digit span. brain function: resting state EEG.
		Comparison *n =* 12	76.7 ± 3.3	75.0	Arm 2 (control): instructed to maintain usual lifestyle for 12 weeks	
Suo et al. (2016) & Broadhouse et al. (2020); Australia^∧^	MCI; Petersen Criteria with MMSE 24-28, CDR <0.5	Intervention *n =* 16	70.1 ± 6.7	68.0	Arm 1 (resistance training): 90 min supervised pneumatic resistance training involving 3 sets of 8 repetitions of 5–6 exercises for major muscle groups, supervised at 80% peak capacity, 2–3 times/week for 26 weeks	Cognition: ADAS-Cog. Brain function: resting state fMRI
		Comparison *n =* 22			Arm 2 (control): 90 min supervised sham training session with stretching, seated calisthenics, and an educational video, 2 times/week for 26 weeks	
Lin et al. (2023); China	Cognitive frailty assessed via EFS ≥5, MoCA <26, GDS ≥2	Intervention *n =* 51	67.7 ± 5.2	62.7	Arm 1 (mind-body exercise): 60 min supervised group Baduanjin 3 times/week, plus 30 min health education every 8 weeks, for 24 weeks	Cognition: C-MoCA. brain function: cerebral blood flow
		Comparison *n =* 51	65.4 ± 5.2	60.8	Arm 2 (control): instructed to maintain usual physical activity and received 30 min health education training once every 8 weeks	
**Three-arm RCTs**						
Nagamatsu et al. (2012); Canada	Probable MCI; Self-reported memory complaints with MoCA <26	Intervention 1 *n =* 30	75.1 ± 3.6	100	Arm 1 (aerobic exercise: 60 min supervised walking at 40% HR reserve and gradually increased to 70–80% of HR reserve, 2 times/week for 24 weeks	Cognition: Trail making test. brain function: task-based fMRI
		Intervention 2 *n =* 28	75.6 ± 3.6	100	Arm 2 (resistance training): 60 min supervised high-intensity strength exercises with a leg-press machine and free weights of 2 sets of 6–8 with loading progressively increased once participants completed the sets with proper form, 2 times/week for 24 weeks	
		Comparison n = 28	73.9 ± 3.4	100	Arm 3 (control): balance and tone exercises involving stretching, range of motion, and relaxation techniques, 2 times/week for 24 weeks	
Tsai et al. (2019); Taiwan	Amnestic MCI; Petersen Criteria with MMSE >24	Intervention 1 *n =* 19	66.0 ± 7.7	73.7	Arm 1 (aerobic exercise): 40 min supervised treadmill or bicycle exercise at 60–75% HR reserve, with 5 min warm-up and cool-down periods, 3 times/week for 16 weeks	Cognition: MMSE, digit span. brain function: event-related potential
		Intervention 2 *n =* 18	65.4 ± 6.8	61.1	Arm 2 (resistance training): 40 min supervised free weight and bodybuilding machine exercises at 60-75% 1RM for 3 sets of 10 repetitions and 90 second inter-set rest and 2 min rest between exercises, with 5 min warm-up and cool-down periods, 3 times/week for 16 weeks	
		Comparison n= 18	65.2 ± 7.0	72.2	Arm 3 (control): supervised stretching with 5 min warm-up and cool-down periods, 3 times/week for 16 weeks	
Liu et al. (2021) and Tao et al. (2019); China†	MCI; Petersen Criteria, with MoCA <26, GDS ≥2	Intervention 1 *n =* 17	64.3 ± 2.6	58.8	Arm 1 (aerobic exercise): 60 min supervised walking at 55-75% of HR reserve 3 times/week, plus 30 min health education every 8 weeks, for 24 weeks	Cognition: C-MoCA. Brain function: resting state fMRI
		Intervention 2 *n =* 20	66.2 ± 4.2	75.0	Arm 2 (mind-body exercise): 60 min supervised group Baduanjin 3 times/week, plus 30 min health education every 8 weeks, for 24 weeks	
		Comparison *n =* 20	66.0 ± 5.7	70.0	Arm 3 (control): instructed to maintain usual physical activity and received 30 min health education training once every 8 weeks, for 24 weeks	
Xia et al. (2019); China	MCI; Petersen Criteria	Intervention 1 *n =* 23	65.8 ± 4.4	73.9	Arm 1 (aerobic exercise): 60 min supervised walking at 55–75% of HR reserve, 3 times/week, plus health education, for 24 weeks	Cognition: Digit symbol. brain function: resting state fMRI
		Intervention 2 *n =* 23	64.9 ± 3.3	52.2	Arm 2 (mind-body exercise): 60 min supervised group Baduanjin, plus health education, 3 times/week for 24 weeks	
		Comparison *n =* 23	65.9 ± 5.3	73.9	Arm 3 (control): instructed to maintain usual physical activity and received 30 min health education training once every 8 weeks, for 24 weeks	

^*^Multi-paper study reporting on different brain function measures from the same intervention. ^∧^Multi-paper study reporting on outcomes from the same intervention at different timepoints. ^†^Multi-paper study reporting on different brain function measures from the same intervention.

ADNI, Alzheimer's Disease Neuroimaging Initiative; CDR, Clinical Dementia Rating; C-MoCA, Chinese-Montreal Cognitive Assessment; EFS, Edmonton Frailty Scale; ERP, Event-Related Potential; fMRI, functional Magnetic Resonance Imaging; GDS, Global Deterioration Scale; HR, Heart Rate; K-MoCA, Korean-Montreal Cognitive Assessment; MCI, Mild Cognitive Impairment; MMSE, Mini-Mental State Exam; MoCA, Montreal Cognitive Assessment; NIAAA, National Institute on Aging and Alzheimer's Association; VCI, Vascular Cognitive Impairment.

### 3.3. Participant characteristics

A total of 646 participants with cognitive frailty, MCI, or VCI were included and their baseline characteristics are summarized in Table 2. For multi-paper interventions, the number of participants from the main study (i.e., Suo et al., 2016; Zhu et al., 2018; Tao et al., 2019) was used in the calculation for total participants included in this review. There were no studies involving people classified with SCD.

Population classification criteria varied between the studies with published guidelines being used in all but one trial (Zhu et al., 2018), and a MoCA or MMSE cut-off score being used in all but two studies (Xia et al., 2019; Yogev-Seligmann et al., 2021). MoCA cut-off scores were consistently applied at ≤ 26 while the MMSE cut-off was inconsistently used: <25 (Amjad et al., 2019), >20 (Hsu et al., 2018), >24 (Suo et al., 2016; Zhu et al., 2018; Qi et al., 2019; Tsai et al., 2019; Tomoto et al., 2021). Mean age ranged from 58 to 78 years, and female representation in samples ranged from 38.4 to 100%.

### 3.4. Intervention characteristics

The last two columns of Table 2 summarize the exercise intervention characteristics and outcome measures relevant to this review. Of the nine two-arm trials, six employed aerobic exercise at a moderate intensity (Hsu et al., 2018; Zhu et al., 2018; Amjad et al., 2019; Qi et al., 2019), two used a resistance training protocol (Suo et al., 2016; Hong et al., 2018), and one implemented the mind-body exercise of Baduajin (Lin et al., 2023). In the three-arm trials, aerobic and resistance training protocols were used in two studies (Nagamatsu et al., 2012; Tsai et al., 2019), while aerobic and mind-body exercise regimes were employed in the other two studies (Tao et al., 2019; Xia et al., 2019). Most interventions were compared to usual care or lifestyle, while three employed sham exercise (Nagamatsu et al., 2012; Suo et al., 2016; Amjad et al., 2019), and a further four involved a concurrent treatment of health education across all groups (Hsu et al., 2018; Tao et al., 2019; Xia et al., 2019; Lin et al., 2023). Exercise frequency ranged from 2 to 3 sessions per week and intervention duration ranged from 6 to 24 weeks, with the most common being 24 weeks.

Supervision was employed in all aerobic exercise trials, and this was conducted through walking (Nagamatsu et al., 2012; Hsu et al., 2018; Xia et al., 2019), dancing (Zhu et al., 2018; Qi et al., 2019), or on a treadmill or stationary bicycle (Amjad et al., 2019; Tao et al., 2019; Yogev-Seligmann et al., 2021) for a duration of 20–60 minutes. Intensity varied between 60–90% of max HR (Zhu et al., 2018; Amjad et al., 2019; Qi et al., 2019; Tomoto et al., 2021) or 50–80% of HR reserve (Nagamatsu et al., 2012; Hsu et al., 2018; Tao et al., 2019; Tsai et al., 2019; Xia et al., 2019; Yogev-Seligmann et al., 2021). Resistance training protocols prescribed exercises at an intensity >65%1RM and adhered to the principles of progressive overload using an elastic band for 40 min (Hong et al., 2018) or a machine and free weights for 40–90 min (Nagamatsu et al., 2012; Suo et al., 2016; Tsai et al., 2019). Three interventions employed the mind-body exercise of Baduanjin for 60 min, three times per week (Tao et al., 2019; Xia et al., 2019; Lin et al., 2023). Seven trials reported warm-up and cool-down periods lasting 5–10 min (Hong et al., 2018; Hsu et al., 2018; Zhu et al., 2018; Amjad et al., 2019; Qi et al., 2019; Tsai et al., 2019; Tomoto et al., 2021; Yogev-Seligmann et al., 2021).

### 3.5. Cognitive outcome measures and effects

**Figure 2:**
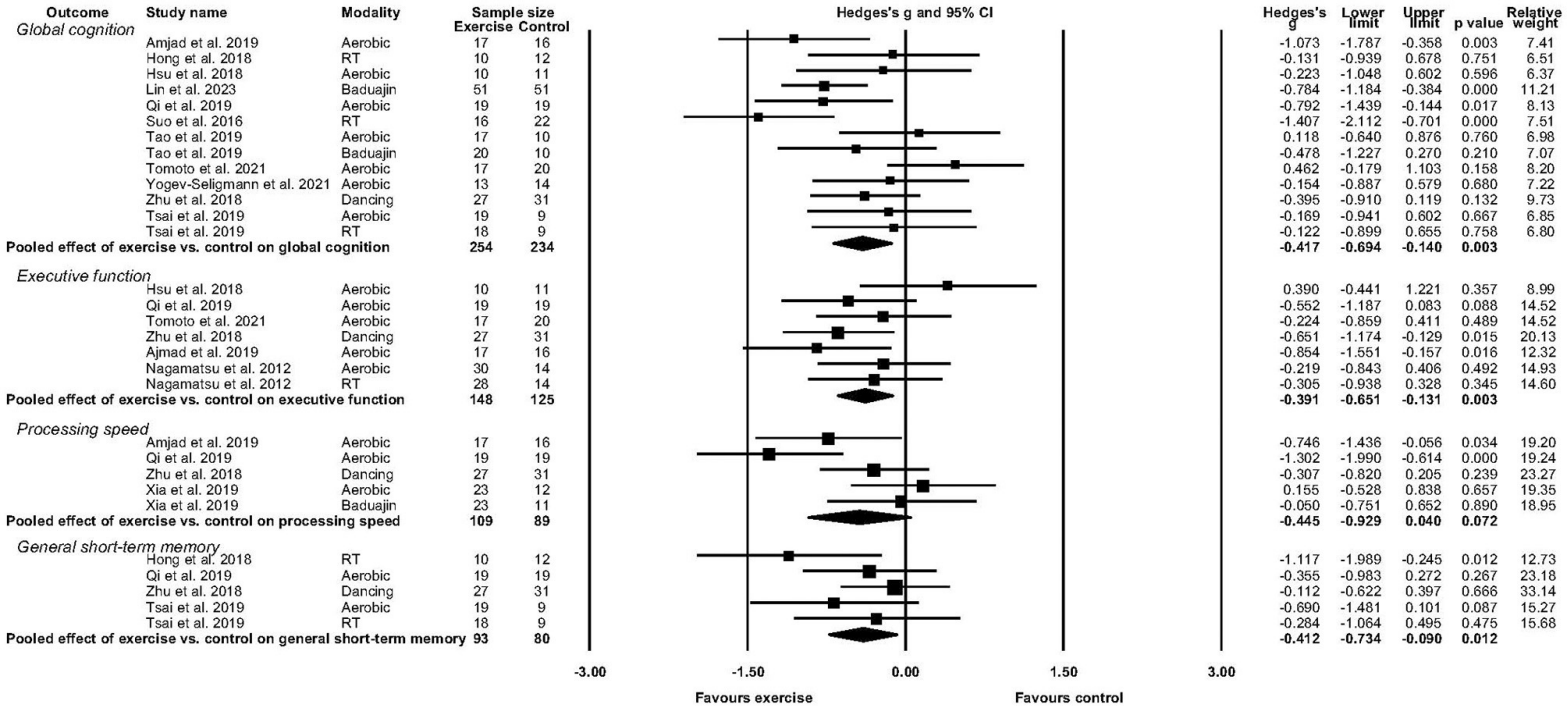
Forest plot depicting effect of exercise versus control on global cognition, executive function, processing speed, and general short-term memory.

As shown in Table 3, four cognitive domains were assessed across the included studies: global cognition (as measured by the MoCA, MMSE, and ADAS-Cog), executive function (most commonly assessed using Trail Making Test B), processing speed (most commonly measured with the Trail Making Test A), and general short-term memory (assessed with Digit Span measures). The pooled effects of exercise vs control groups for these cognitive outcomes are summarized in Figure 2.

**Table 3:** Domain-specific cognitive performance outcomes of exercise and control groups from included trials.

	**Cognitive performance outcomes**					
	**Exercise pre mean (SD)**	**Exercise post mean (SD)**	**Control pre mean (SD)**	**Control post mean (SD)**	**Exercise pre/post change mean (SD)**	**Control pre/post change mean (SD)**
**Global cognition**						
Amjad et al., MoCA	18.88 (1.17)	22.88 (1.65)	19.76 (1.64)	20.94 (3.27)	N/A	N/A
Hong et al., K-MoCA	20.70 (3.46)	21.70 (3.05)	20.08 (4.44)	20.50 (5.05)	N/A	N/A
Hsu et al., MoCA	22.20 (2.40)	22.30 (1.40)	24.10 (2.10)	23.60 (3.30)	N/A	N/A
Lin et al., C-MoCA	22.67 (2.83)	N/A	21.55 (3.67)	N/A	2.51 (2.29)	0.34 (3.14)
Qi et al., MoCA	22.60 (2.10)	24.30 (2.20)	23.70 (1.70)	23.70 (2.00)	N/A	N/A
Suo et al., ADAS-Cog	N/A	N/A	N/A	N/A	−2.15 (0.96)	−0.93 (0.76)
Tao et al., C-MoCA	AE 21.47 (2.27)	N/A	21.00 (2.36)	N/A	0.88 (1.96)	1.10 (1.48)
	BDJ 22.45 (2.16)	N/A			2.10 (2.25)	
Tomoto et al., MMSE	29.2 (1.36)	28.5 (1.46)	28.5 (1.42)	28.5 (1.50)	N/A	N/A
Tsai et al., MMSE	AE 27.16 (1.26)	AE 27.26 (1.10)	27.00 (1.65)	26.89 (1.41)	N/A	N/A
	RT 26.56 (1.34)	RT 26.61 (1.20)			N/A	
Yogev-Seligmann et al., MoCA	23.7 (2.72)	22.9 (4.17)	26.0 (2.25)	24.6 (3.37)	N/A	N/A
Zhu et al., MoCA	23.20 (1.90)	24.70 (2.20)	22.90 (2.10)	23.60 (1.80)	N/A	N/A
**Executive Function**						
Amjad et al., TMT-B	221.40 (61.20)	168.00 (69.60)	228.6 (75.60)	228.66 (50.40)	N/A	N/A
Hsu et al., Flanker IRT	737.90 (119.70)	708.00 (74.40)	823.70 (109.70)	764.4 (70.50)	N/A	N/A
Nagamatsu et al., TMT B minus A	N/A	N/A	N/A	N/A	AE 8.83 (41.86)	−0.39 (40.27)
	N/A	N/A			RT 9.13 (19.88)	
Qi et al., TMT-B	190.60 (59.20)	161.60 (53.80)	182.20 (57.70)	181.60 (46.70)	N/A	N/A
Tomoto et al., D-KEFS TMT	11.4 (1.78)	11.8 (1.71)	11.7 (1.84)	12.5 (1.78)	N/A	N/A
Zhu et al., TMT-B	200.00 (73.00)	158.00 (49.00)	187.00 (67.00)	177.00 (48.00)	N/A	N/A
**Processing Speed**						
Amjad et al., TMT-A	128.40 (45.60)	84.60 (39.00)	141.00 (55.20)	132.00 (51.60)	N/A	N/A
Qi et al., TMT-A	107.30 (97.10)	71.00 (29.30)	72.20 (23.30)	68.80 (19.10)	N/A	N/A
Xia et al., Digit Symbol	AE 34.65 (10.14)	AE 37.59 (6.14)	31.89 (7.27)	36.02 (9.69)	N/A	N/A
	BDJ 33.22 (10.63)	BDJ 37.72 (5.85)			N/A	
Zhu et al., TMT-A	74.00 (29.00)	66.00 (25.00)	70.00 (23.00)	69.00 (20.00)		
**General Short-Term Memory**						
Hong et al., Digit Span Backward	2.32 (1.28)	2.17 (1.52)	2.50 (0.97)	1.08 (0.91)	N/A	N/A
Qi et al., Digit Span Backward and Forward	16.40 (2.90)	16.40 (2.60)	18.10 (3.40)	17.10 (2.90)	N/A	N/A
Tsai et al., Digit Span	AE 19.00 (1.67)	AE 19.32 (1.77)	19.83 (2.23)	18.78 (2.24)	N/A	N/A
	RT 20.33 (1.85)	RT 19.89 (2.00)			N/A	
Zhu et al., Digit Span Backward and Forward	16.80 (2.70)	16.90 (2.30)	17.20 (2.90)	17.00 (2.90)	N/A	N/A

AE, Aerobic Exercise; BDJ, Baduanjin; IRT, Incongruent trial Reaction Time; K-MoCA, Korean-Montreal Cognitive Assessment; MoCA, Montreal Cognitive Assessment; N/A, Not Available; RT, Resistance Training; TMT-A, Trail Making Test A; TMT-B, Trail Making Test B.

#### 3.5.1. Global cognition

Eleven studies reported sufficient data to determine the pooled effect of exercise vs. control for change in global cognition (Suo et al., 2016; Hong et al., 2018; Hsu et al., 2018; Zhu et al., 2018; Amjad et al., 2019; Qi et al., 2019; Tsai et al., 2019; Tomoto et al., 2021; Yogev-Seligmann et al., 2021; Lin et al., 2023). Exercise elicited a medium and significant improvement in global cognition (g = −0.417, 95% CI, −0.694 to −0.140, *p* = 0.003, *I*^2^ = 43.56%, τ =0.369, n =488). There was no indication of publication bias (p = 0.246).

#### 3.5.2. Executive function

Six studies reported sufficient data to determine the pooled effect of exercise vs. control for change in executive function (Nagamatsu et al., 2012; Hsu et al., 2018; Zhu et al., 2018; Amjad et al., 2019; Qi et al., 2019; Tomoto et al., 2021). Exercise elicited a medium and significant improvement in executive function (g = −0.391, 95% CI, −0.651to −0.131, *p* = 0.003, *I*^2^ = 13.28%, τ =0.185, n =273). There was no indication of publication bias.

#### 3.5.3. Processing speed

Four studies reported sufficient data to determine the pooled effect of exercise vs control for change in processing speed (Zhu et al., 2018; Amjad et al., 2019; Qi et al., 2019; Xia et al., 2019). Exercise elicited a medium but non-significant change in processing speed (g = −0.445, 95% CI, −0.929 to 0.040, *p* = 0.072, *I*^2^ = 64.11%, τ =0.441, *n* =198). There was no indication of publication bias.

#### 3.5.4. General short-term memory

Four studies reported sufficient data to determine the pooled effect of exercise vs. control for change in short-term memory performance (Hong et al., 2018; Zhu et al., 2018; Qi et al., 2019; Tsai et al., 2019). Exercise elicited a medium but non-significant improvement in short-term memory function (g = −0.412, 95% CI, −0.734 to 0.090, *p* = 0.012, *I*^2^ = 10.01%, τ =0.118, *n* =173). There was no indication of publication bias.

### 3.6. Brain function

#### 3.6.1. fMRI

Eight fMRI studies were identified, with three studies each assessing hemodynamic activity during cognitive tasks: associative memory (Nagamatsu et al., 2012; Yogev-Seligmann et al., 2021), complex information processing (Yogev-Seligmann et al., 2021), and flanker tasks (Hsu et al., 2018), and the other five utilizing an eyes closed resting state (Suo et al., 2016; Qi et al., 2019; Tao et al., 2019; Xia et al., 2019; Liu et al., 2021).

In an associative memory task (Nagamatsu et al., 2012), 6-months resistance training altered hemodynamic activity in the right lingual gyrus (*p* = 0.03), occipital-fusiform gyrus (*p* = 0.02), and right frontal pole (*p* = 0.03) during the encoding and recall of associations, when compared with the control group (direction of effects not reported). Hemodynamic changes in the right lingual gyrus correlated positively with memory performance (*r* = 0.51, *p* = 0.02); while aerobic training effects were comparable to the control group (Nagamatsu et al., 2012). During the encoding phase of another associative memory task, 16-weeks aerobic exercise increased BOLD activity in frontal regions including left inferior frontal gyrus (*p* < 0.001), left precentral gyrus (*p* =0.001), and left middle frontal gyrus (*p* =0.044) in people with amnestic MCI (Yogev-Seligmann et al., 2021). There was also aerobic exercise-related enhanced response synchronization in the supramarginal gyrus, temporo-parietal junction, inferior frontal gyrus, middle frontal gyrus, insular cortices, anterior cingulate, precuneus and cuneus in their complex information processing paradigm (Yogev-Seligmann et al., 2021). In the flanker task (Hsu et al., 2018), 6-months aerobic training reduced activity in the left lateral occipital cortex (*p* < 0.03) and the right superior temporal gyrus (*p* = 0.03), relative to the control group. These reductions were significantly associated with faster reaction times on congruent trials (both *p* = 0.04). The decrease in superior temporal gyrus activity also correlated with better performance on incongruent trials (*p* = 0.05) (Hsu et al., 2018).

Of the five resting state studies, one reported significant between-groups effects (Xia et al., 2019), with 24-weeks Baduanjin reducing functional connectivity in the right supramarginal inferior parietal and angular gyri, right rolandic operculum, right precuneus, and right fusiform gyrus when compared with the control group (all *p* < 0.05). Both exercise groups (Baduanjin and brisk walking) led to reduced functional connectivity in the right middle temporal gyrus relative to controls (all *p* ≤ 0.042). However, these functional connectivity changes were not associated with Stroop task performance (all *p* > 0.05). Another Baduanjin intervention study (Liu et al., 2021) assessing people with MCI found that 6-months training increased resting state functional connectivity from the right locus coeruleus and left ventral tegmental area to the right insula, right amygdala, and right anterior cingulate. The increased connectivity between the right locus coeruleus, the right insula (*r* =0.277, *p* =0.037), and the right anterior cingulate (*r* =0.265, *p* =0.046) were correlated with global cognitive performance (MoCA) (Liu et al., 2021). Progressive resistance training (Suo et al., 2016) for 6-months in people with MCI decreased functional connectivity between the posterior cingulate and both the left inferior temporal lobe and anterior cingulate cortex, and between the bilateral hippocampi and the right inferior temporal lobe (all *p* < 0.001). Functional connectivity was increased between the bilateral hippocampi and right middle frontal lobe (*p* =0.001). These changes in functional connectivity were not associated with the improvements in cognition observed with the resistance training intervention (Suo et al., 2016).

Within-group analyses in another study (Qi et al., 2019) showed that 3-months aerobic exercise increased the amplitude of low-frequency fluctuations (ALFF) in bilateral fronto-temporal, entorhinal, anterior cingulate and parahippocampal cortices (all *p* < 0.05); whereas controls exhibited increased ALFF in the right temporal and posterior cingulate cortex (*p* < 0.05). These changes in ALFF were not related to cognitive test performance. Another sub-study (Tao et al., 2019) from the trial assessing 24-weeks Baduanjin training in people with cognitive frailty found that Baduanjin training led to significant ALFF reductions in the right hippocampus (classic low-frequency band 0.01–0.08 Hz) and increases in the bilateral anterior cingulate (slow-5 band 0.010–0.027 Hz); these were linked with MoCA score changes (hippocampus: *r* = −0.291, *p* =0.036; anterior cingulate: *r* =0.309, *p* =0.025).

#### 3.6.2. Electrophysiology

Four studies examined electrophysiological activity (Hong et al., 2018; Zhu et al., 2018; Amjad et al., 2019; Tsai et al., 2019). There were two EEG studies identified, with neuronal activity assessed in the delta to beta band ranges during alternating eyes closed and open states in one study (Amjad et al., 2019) and eyes closed in the second study (Hong et al., 2018). Relative eyes closed resting state power in delta and alpha-2 were lower, beta-1 was higher, and EEG complexity (approximate entropy) increased after 6-weeks (18 sessions) aerobic exercise compared with controls (all *p* < 0.05). There were no significant differences in the eyes-open condition (Amjad et al., 2019). In the second study, relative theta power in the frontal-left region decreased, while relative alpha power in the temporal-left region increased with 12-weeks resistance training as compared to the control group (both *p* < 0.05) (Hong et al., 2018).

Two ERP studies were found (Zhu et al., 2018; Tsai et al., 2019). In the one auditory task ERP study of people with MCI (task details not described; Zhu et al., 2018) that assessed P300 component amplitudes and latencies, 3-months of aerobic exercise (dancing) reduced P300 component latency relative to controls, and this effect was sustained at the 6-month follow-up (both *p* < 0.05). In the second ERP study (Tsai et al., 2019), 16-weeks exercise in people with amnestic MCI increased P3 amplitudes from a visual task switching paradigm following both aerobic (*p* < 0.001) and resistance training (*p* = 0.013) compared to controls.

#### 3.6.3. Cerebral blood flow

Two studies assessed cerebral blood flow (Tomoto et al., 2021; Lin et al., 2023). Tomoto et al. (2021) measured cerebral blood flow with transcranial doppler in people with MCI. Following 12-months aerobic exercise training, internal carotid arterial flow (*p* = 0.023) and normalized cerebral blood flow (*p* = 0.006) increased, and in the middle cerebral artery, diastolic cerebral blood flow velocity (*p* = 0.020) increased, and pulsatility index deceased (*p* =0.030). These changes were not associated with cognitive performance (Tomoto et al., 2021).

The 24-week trial of Baduanjin in people with cognitive frailty (Lin et al., 2023) led to increased blood flow velocity in the right middle cerebral artery and basilar artery, and increased end diastolic velocity to the basilar artery; with a decrease reported for the control group (all *p* < 0.023). Reductions in peak systolic velocity in the bilateral middle cerebral artery were observed for both Baduanjin training and the control group (all *p* < 0.045); these were lower for controls. Correlations with cognitive function were not explored (Lin et al., 2023).

### 3.7. Methodological quality

The results of the methodological quality assessment are presented in Table 4. The scores ranged from 13–20, with an average of 16.9. Seven studies were classified as being of moderate quality (Hong et al., 2018; Zhu et al., 2018; Amjad et al., 2019; Tao et al., 2019; Tsai et al., 2019; Tomoto et al., 2021; Yogev-Seligmann et al., 2021), and one was considered to be of high quality (Hsu et al., 2018). All studies did not blind their intervention groups due to the nature of the exercise trials. Most articles (*n* = 11) adequately reported their aims, outcomes, patient characteristics, interventions, *p*-values, and variabilities.

**Table 4:** Results of the modified methodological quality assessment (Downs and Black, 1998).

**Author**	**Reporting** ^**$**^										**External Validity** ^*^			**Internal validity**										**Score**
														**Bias** ^α^				**Confounding** ^β^						
	**01**	**02**	**03**	**04**	**05**	**06**	**07**	**08**	**09**	**10**	**11**	**12**	**13**	**15**	**18**	**19**	**20**	**21**	**22**	**23**	**26**	**27**	**28**	**/23**
Amjad et al. (2019)	1	1	1	1	1	1	1	0	0	1	1	1	1	1	1	0	1	1	0	1	0	0	1	17
Hong et al. (2018)	1	1	1	1	1	1	1	0	1	1	1	1	1	0	1	0	1	1	0	1	1	0	1	18
Hsu et al. (2018)	1	1	1	1	1	1	1	0	0	1	1	1	1	1	1	1	1	1	1	1	1	0	1	20
Lin et al. (2023)	1	1	1	1	1	1	1	0	0	1	1	1	0	1	1	0	1	0	1	1	0	0	1	16
Liu et al. (2021)^*^	1	1	1	1	1	1	1	0	1	1	0	0	0	0	1	1	1	1	0	1	1	0	1	16
Nagamatsu et al. (2012)	1	1	1	1	1	1	0	1	0	1	0	0	0	0	1	1	1	0	0	1	0	0	1	13
Qi et al. (2019)	1	1	1	1	1	1	1	0	1	1	0	0	0	1	1	0	1	0	1	1	1	0	1	16
Suo et al. (2016)	1	1	1	1	0	1	1	0	0	1	0	0	0	1	1	1	1	1	0	1	1	0	1	15
Tao et al. (2019)^*^	1	1	1	1	1	1	1	0	1	1	1	1	0	0	1	1	1	1	0	1	1	1	1	19
Tomoto et al. (2021)	1	1	1	1	1	1	1	0	0	1	1	1	1	1	1	1	1	1	0	1	0	0	0	17
Tsai et al. (2019)	1	1	1	1	1	1	1	0	1	1	1	1	1	0	1	1	1	1	0	1	1	0	1	19
Xia et al. (2019)	0	1	1	1	1	1	1	0	0	1	1	1	1	0	1	0	1	1	0	1	0	0	1	15
Yogev-Seligmann et al. (2021)	1	1	1	1	1	1	1	0	1	1	1	1	1	0	1	1	1	1	0	1	1	0	1	19
Zhu et al. (2018)	1	1	1	1	1	1	1	1	1	1	0	0	1	1	1	0	1	0	1	1	0	0	1	17

^$^Reporting category includes items such as, study aims, reported outcomes, patient characteristics, confounders, adverse events and loss to follow-up.

^*^External validity includes questions regarding to the study population.

^α^Internal validity - bias includes items such as blinding, follow-up and compliance.

^β^Internal validity - confounding includes items such as study selection, randomization and study power.

Six trials reported the adherence to exercise sessions, with an average adherence rate of ≥54% (Nagamatsu et al., 2012; Suo et al., 2016; Hsu et al., 2018; Tao et al., 2019; Tsai et al., 2019; Tomoto et al., 2021; Yogev-Seligmann et al., 2021). Furthermore, all included trials provided supervised exercise sessions. Three studies reported adverse effects, which included acute episodes of shortness of breath and non-injurious falls in one study (Nagamatsu et al., 2012), and no adverse effects in the other studies (Zhu et al., 2018; Yogev-Seligmann et al., 2021). Seven studies reported attempted to blind those measuring cognitive outcomes of the exercise interventions (Suo et al., 2016; Hsu et al., 2018; Zhu et al., 2018; Amjad et al., 2019; Qi et al., 2019; Tomoto et al., 2021; Lin et al., 2023).

### 3.8. Risk of bias

**Figure 3:**
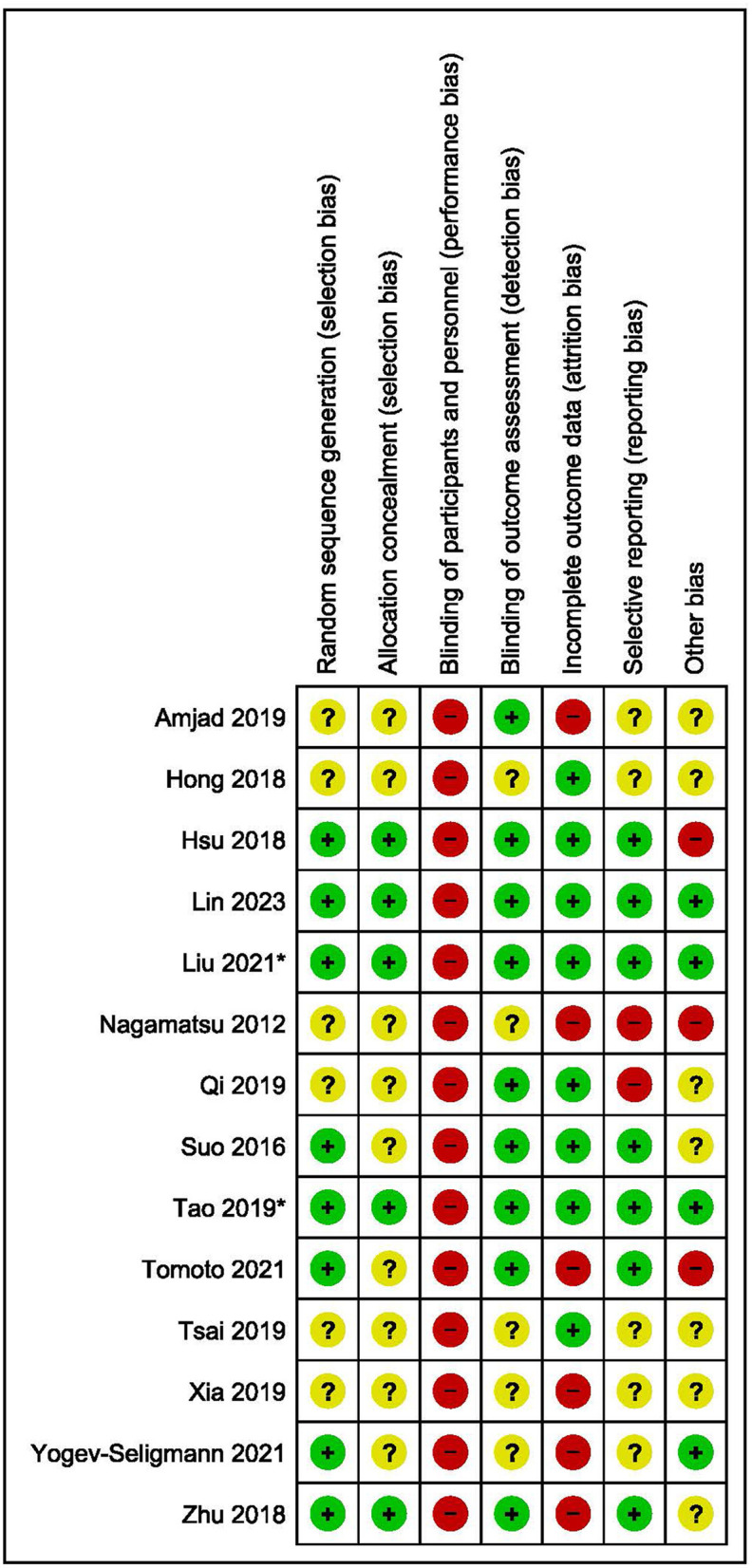
Risk of bias assessment summary.

The results of the risk of bias assessment are summarized in Figure 3. Five studies scored an unclear or high risk of bias on six or more domains (Nagamatsu et al., 2012; Hong et al., 2018; Amjad et al., 2019; Tsai et al., 2019; Xia et al., 2019). Two studies scored an unclear or high risk of bias on five domains (Qi et al., 2019; Yogev-Seligmann et al., 2021), while six studies scored an unclear or high risk of bias on four or less domains (Suo et al., 2016; Hsu et al., 2018; Zhu et al., 2018; Tao et al., 2019; Tomoto et al., 2021; Lin et al., 2023).

## 4. Discussion

This systematic review aimed to synthesize and evaluate exercise interventions for effects on cognition and brain function in adults with cognitive impairment. As evidenced by our screening outcomes (see Figure 1), this literature base was rather limited and is considerably novel, with fifteen articles identified and thirteen of these being published within the last 4 years (since 2018). All but two studies included people with MCI (Hsu et al., 2018; Lin et al., 2023) and all but one referred to published guidelines in their classification criteria (Nagamatsu et al., 2012). No studies involving people with SCD met eligibility criteria. Intervention protocols mostly involved moderate intensity aerobic exercise and were conducted 2–3 times per week for up to 40 MIN per session. Most studies assessed global cognition using the MoCA and executive functions through the Trail Making Test B, with most brain function assessments conducted using fMRI.

### 4.1. Exercise and cognition

According to our analyses, exercise leads to moderate and significant improvements in global cognitive and executive functions, and these are driven by interventions of aerobic nature. The cognitive benefits of aerobic exercise (high- and low-frequency) have been reported in previous meta-analyses and reviews extending to those who have AD and non-AD dementias, MCI, and SCD (Baker et al., 2010; Groot et al., 2016; Zheng et al., 2016; Cammisuli et al., 2017). Studies utilizing strength-based exercise for cognition in older adults, however, are lacking. Only four studies included in this meta-analysis employed resistance strength-based training. Further, none of the studies explored the effects of resistance training on processing speed. Reduced muscular strength has been associated with advancing age (Lauretani et al., 2003), greater brain atrophy, white matter disease, and poorer cognitive function (Clark and Taylor, 2011; Kilgour et al., 2014; Herold et al., 2019).

Growing evidence shows that resistance exercise evokes meaningful benefits in functional brain changes (particularly the frontal lobe), reductions in white matter lesions and decreased atrophy across age-groups (Herold et al., 2019). For example, in older sedentary males, a 6-month study presented that resistance training improved memory performance (Cassilhas et al., 2007). In community-dwelling older women, progressive resistance exercise significantly improved executive function by up to 12.6% (Liu-Ambrose et al., 2010). Further, at 2-year follow-up within that same cohort, both bi-weekly and weekly resistance exercise increased peak muscle power, maintained executive functions, promoted memory, and reduced white matter atrophy (Best et al., 2015).

In an MCI population that compared progressive resistance training (PRT) with computerized cognitive training (CCT), the authors found that the PRT but not CCT significantly increased gray matter in the posterior cingulate, reduced white matter hyperintensity, and improved global cognition at 6-months follow-up (Suo et al., 2016). From our analyses, pooled effects for short-term memory were moderate, but non-significant. However, these marginal improvements in short-term memory seem to be driven by resistance training, rather than aerobic exercises, which has not been reported elsewhere. Together, this suggests that resistance exercise has cognitive benefits independent of aerobic exercise. Previous research has demonstrated plausible biological mechanisms involving homocysteine and insulin-like growth factor 1 (Liu-Ambrose and Donaldson, 2009). As an interesting aside, it has been claimed by an older meta-analysis that the greatest benefits on cognition in older adults could occur when aerobic exercise was combined with resistance training (Colcombe and Kramer, 2003). Regardless, more clinical trials examining the role of resistance strength-based exercise independently or combined with aerobic exercises are required to explore the maximal benefits on cognitive functions and associated physiological changes.

### 4.2. Exercise and brain function

Regional differences throughout the brain were reported across studies utilizing fMRI to assess central mechanisms of exercise, with few commonalities (in study design and subsequently results), making synthesis of results challenging. Task-based fMRI assessments revealed exercise-related changes in distinct brain areas, with two of the three studies respectively correlating right lingual gyrus and superior temporal gyrus changes with improved cognitive performance (Nagamatsu et al., 2012; Hsu et al., 2018). The most pronounced resting state changes observed were in the temporal lobes, with 12–26 weeks of aerobic, resistance, and mind-body exercises altering the hippocampal region (Suo et al., 2016; Qi et al., 2019; Tao et al., 2019). While most studies found non-significant correlations between the regional changes in brain activity and cognitive performance (Suo et al., 2016; Qi et al., 2019; Xia et al., 2019), improved MoCA scores were associated with Baduanjin-related changes in resting state anterior cingulate activity (Tao et al., 2019; Liu et al., 2021). These findings are likely due to increased regional metabolic activity (Shimada et al., 2017) and may reflect exercise-induced neuroplastic changes as observed in other populations (Woodward et al., 2020), potentially providing a buffer against pathophysiological and age-related atrophy (Bugg and Head, 2011) in these vulnerable regions.

In AD, the resting state EEG signature is dominated by slow wave activity (i.e., increased delta and theta magnitude) and reduced fast wave activity (alpha and beta magnitude) (Babiloni et al., 2021), and a similar pattern has been reported in MCI (Meghdadi et al., 2021) (albeit less consistently), particularly those who have an increased risk of AD (Jelic et al., 2000). However, in healthy aging, the opposite is observed, that is, reduced delta and theta spectral power (Vlahou et al., 2014; Barry and De Blasio, 2017). Across included studies, 12–16 weeks of exercise (both aerobic and resistance) led to reduced delta and theta power in MCI (Hong et al., 2018; Amjad et al., 2019), potentially indicating a normalization of the EEG spectra and a shift away from the AD trajectory. Exercise differentially modulated alpha, with aerobic training reducing alpha-2 power (11–14 Hz) (Amjad et al., 2019) and resistance training increasing broad-band alpha (8–12 Hz) power (Hong et al., 2018). Hong's finding may have more therapeutic relevance here given that reduced alpha power (and peak frequency) is reported in MCI/AD, and the wider alpha range may have detected this. Further, aerobic exercise appears to promote neural adaptability that is measurable at a global scale. Aerobic exercise increased EEG complexity (Amjad et al., 2019), a signal proposed to reflect the underlying integrity of neuronal circuitry and its capacity to rapidly adapt to environmental changes (Bosl et al., 2011). This is further reflected in the ERP effects of aerobic and resistance exercise whereby reduced P300 latency and increased P300 amplitude indicated faster stimulus evaluation processing and enhanced neurocognitive efficiency (Steiner et al., 2013; Zhu et al., 2018; Tsai et al., 2019).

Increased CBF has been suggested as a potential mechanism underpinning the positive effects of exercise on cognitive function (Renke et al., 2022). The exact reason for this is less clear but these effects may arise from the association between neuronal metabolism and CBF and perfusion (i.e., neurovascular coupling), changes in cerebral blood vessels including elasticity and density (i.e., angiogenesis), and enhanced activity of pericytes (Bolduc et al., 2013; Barnes et al., 2021). From the two CBF studies identified in this review, aerobic exercise was found to increase blood flow velocity in the internal carotid artery (Tomoto et al., 2021), right middle cerebral and basilar arteries (Lin et al., 2023), and end diastolic velocity in the middle cerebral artery (Tomoto et al., 2021) and basilar artery (Lin et al., 2023). In the middle cerebral artery, reductions were also seen in pulsatility (Tomoto et al., 2021) and peak systolic velocity (Lin et al., 2023). No associations were found with cognitive function were found in the one study that assessed for them (Tomoto et al., 2021) so we are currently unable to infer whether these changes in arterial CBF are associated with cognitive improvements related to exercise.

### 4.3. Limitations

A small number of studies met eligibility criteria pertaining to intervention characteristics and outcome measures, limiting the implications of this review. Most notably, there were no SCD studies. This is unsurprising given that classification criteria were operationalized in 2017 (Molinuevo et al., 2017), just as brain function measures emerged as outcomes of interest in exercise trials. This limited the scope of our review, its findings, and implications to objective classifications of cognitive impairment, which are respectively considered to represent the prodromal stages of AD and vascular dementia. These findings were also hindered by the small sample sizes, lack of reported adherence to exercise sessions (and where this was reported in two studies, the average adherence rate was 75%), moderate methodological quality, and high risk of bias in most included studies. We also did not exclude studies based on intervention from any pooled analysis. The pooled analyses were undertaken to determine the effect of exercise per se (which can include aerobic exercise or resistance training), to maximize the sample size. Further studies can explore the relative effects of individual exercise prescription variables such as modality, intensity, and volume. These review outcomes speak to the paucity and novelty of brain function research regarding exercise for dementia prevention. Any neuronal mechanistic inferences regarding exercise and cognition are thus speculative.

### 4.4. Conclusion

**Figure 4:**
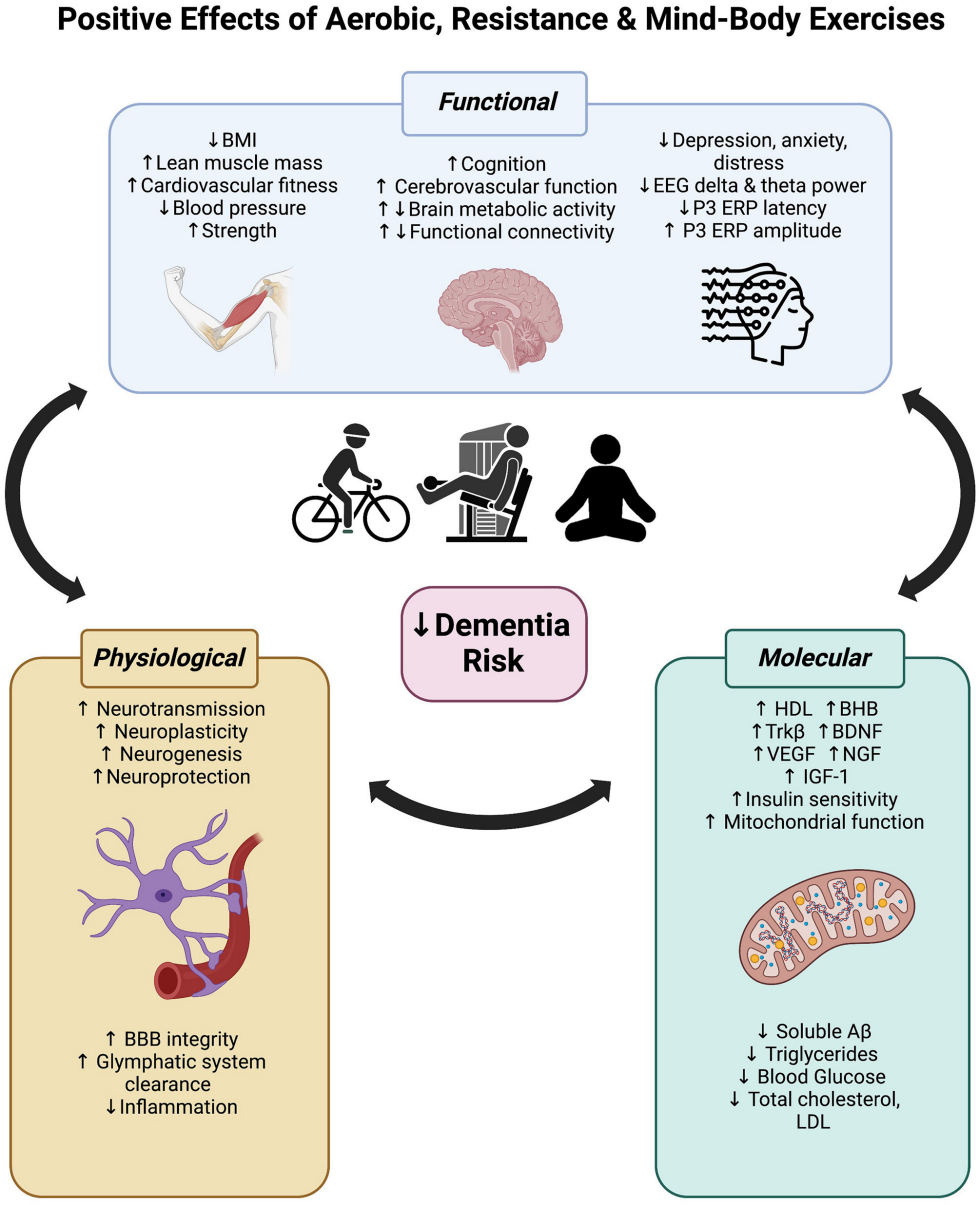
Positive effects of aerobic, resistance and mind-body exercise on functional (body and brain), physiological and molecular mechanisms that may reduce dementia risk.

As reviewed here, exercise can significantly improve global cognitive and executive functions in people with cognitive impairment and increased dementia risk. Regardless of modality (aerobic, resistance, or mind-body), at least 12 weeks of exercise can reverse brain activity signatures of cognitive decline in people with cognitive impairment. We speculate that the cognitive improvements associated with exercise are likely driven by increased metabolic activity, cerebrovascular mechanisms (such as neurovascular coupling), and neuroplasticity throughout the brain, but particularly in highly sensitive and plastic regions in the frontal and temporal lobes. These hypotheses require further investigation with higher quality randomized controlled trials. Figure 4 integrates these central impacts of exercise alongside other functional changes together with molecular and physiological mechanisms established in prior reviews (mentioned in the introduction), highlighting the multitargeting effects of exercise for dementia risk reduction. As the field of dementia prevention grows, we encourage researchers to include more clinical trials investigating the role of resistance strength-based exercise independently or combined with aerobic exercises in this at-risk population. This is to identify the maximal benefits of different exercise regimes on cognition and physiological changes. Furthermore, as highlighted here, future research should integrate both cognitive and brain function measures to help elucidate the mechanisms underpinning the positive effects of exercise.

## Data availability statement

The original contributions presented in the study are included in the article/supplementary material, further inquiries can be directed to the corresponding author.

## Author contributions

DK, AS, and GS-L conceptualized the study. AS and DK conducted the searches while RC and N-JM screened articles. AB, VU, and TT assisted with data extraction. AS and DK conducted the analyses with GS-L and IL interpreting the findings. All authors contributed to the drafting of the manuscript and approved its final form.
